# Immigrant Vietnamese women’s adaptation to culture and society in rural areas of Korea

**DOI:** 10.1371/journal.pone.0212265

**Published:** 2019-02-14

**Authors:** Misoon Jeon, Okhee Ahn, Minjeong An

**Affiliations:** 1 Department of Nursing Science, Baekseok University, Cheonan, Korea; 2 College of Nursing, Woosuk University, Wanju, Korea; 3 College of Nursing, Chonnam National University, Gwangju, Korea; Sogang University (South Korea), REPUBLIC OF KOREA

## Abstract

**Background:**

International marriages between Vietnamese women and Korean men have increased rapidly in Korea. Successful adaptation of these women is challenging, and concerns on the issue have been raised. Most existing studies have surveyed Vietnamese immigrant women in urban Korea; less is known about women residing in rural areas of Korea. Uncovering the experiences and perspectives of these women can inform the design of community support resources. The purpose of this study was to identify a typology to effectively describe the subjective perspectives of Vietnamese women residing in rural Korea on adaptation to Korean culture and society.

**Methods and results:**

A Q-methodological study was conducted with five steps: construction of the concourse, Q sample, selection of participants, Q-sorting, and data analysis. Twenty-six Vietnamese women married to Korean men who attended local public health centers were surveyed and asked to rank-order 39 Q-statements using a 9-point scale. Principal component factor analysis using a pc-QUANL program was performed to identify adaptation sub-types. Four types of adaptation were identified and labeled: positive adaptation (35.54%), passive adaptation with reservations (8.33%), adaptation with frustration (6.20%), and adaptation and involvement (5.33%). These four types explained 55.40% of the total variance in the women’s experience of adaptation to Korean culture and society.

**Conclusions:**

This study provides data that may be helpful in understanding the challenges immigrant women in rural areas of Korea face, and in planning family-sensitive adaptation support programs for these women and their families.

## Introduction

Because many young people have left rural areas and gone to cities seeking jobs and opportunities for a better life, population decline in rural areas of Korea (e.g., agricultural regions and fishing villages) is significant, averaging 5.6% annually from 1980 to 2013 [1]. Consequently, many men, in particular those 35 years and older, face a shortage of potential spouses in these rural areas [2]. Some of them have tried to find eligible partners through international marriage. Local governments, especially in agricultural and fishing areas, facilitate such marriages through the international marriage immigrant support act and policies [3–5]. The act and policies aim to help International Marriage Immigrant Women (IMIW) settle and integrate well in Korea, focusing on issues of human rights, adaptation support, health insurance, and financial assistance to protect and support them and maintain community populations.

### Current status of Vietnamese IMIW in Korea

According to Statistics Korea for 2018, the number of international marriages between Korean men and foreign women has increased significantly from 6,945 in 2000 to a peak of 30,719 in 2005; although the number has fluctuated and decreased since 2006, it remained high in 2017 at 14,869 [6]. In rural areas, international marriages account for approximately a fifth of all marriages (18.4%) in 2017 [7]. Immigrant women married to Korean men are of various Asian heritages; Vietnamese IMIW make up 36.1% of women coming to Korea. This is the largest group, followed closely by Chinese IMIW (26.1%) [6]. In 2017, divorce rates among Vietnamese IMIW ranked second highest, exceeded only by divorce rates among Chinese IMIW [8]. Compared to IMIW of six other nationalities, Vietnamese IMIW were reported to have the second lowest life satisfaction, following Cambodian IMIW [9]. In similar circumstances, Vietnamese IMIW in Taiwan were reported to experience physical and psychological distress in response to adaptation difficulties [10]. Such findings raise concern and suggest that these IMIW and their families may not be adapting as well as other immigrant marriage families.

### Adaptation difficulties among IMIW

Several potential stressors may result in poorer adaptation among IMIW. Many of these women marry with insufficient or distorted information about Korea and their matched husbands [11, 12]. According to study findings on immigrant women in multicultural families, some foreign wives have reported disappointment after marriage because the reality is far different from their expectations [9, 12]. Although some information about their prospective husbands is available in advance (e.g., physical appearance, age, and overall household income), differences in culture and the realities of women’s situations after marriage are potential sources of significant stress that may lead to deterioration of their physical and mental health and poor quality of life [13–15].

Recognizing the potential for maladaptation among immigrant wives, the Korean government and private institutions have endeavored to promote positive adaptation to Korean society by providing a variety of support systems (e.g., Korean language school, pregnancy and childbirth care, job education, and counseling services) [3, 4]. Despite these efforts, the process of adaptation to a new culture or society is complex, and may not be easily improved by these support mechanisms. Previous studies and models may not have adequately informed the design of support programs for these women, particularly IMIW residing in rural areas of Korea.

### Acculturation model and adaptation typologies

Researchers have applied acculturation models to explain the process of cultural adaptation and to predict outcomes; however, these models can fall short. When applied to specific populations, the phenomena of acculturation and adaptation must take into consideration that the process is a personalized one. Women’s experiences cannot be easily grouped or typed without risk of missing the in-depth meaning and context of their experience. Further, older models, such as that by Berry [16], are problematic because they are anchored in a specific time and population. Berry’s model addressed the phenomena of acculturation and adaptation in immigrants, refugees, and asylum seekers who settled in North America and Western Europe. In our study, there are limitations to using an acculturation model that was designed to describe the experience of a different population during a different time period and different context. The model may not adequately capture the circumstances experienced by IMIW in Asia or the experiences of women coming to Korea, particularly those settling in rural areas of the country. Specifically, the population in our study has some unique characteristics; through brokered marriage, women from a lower-income country marry men with an average age difference of about 10 years from a higher-income country with an ethnically homogeneous national society [17]. Under these circumstances, we would expect to find different challenges to successful adaptation. For these reasons, applying a specific conceptual model to the observations of the study participants is problematic and increases the risk of missing the uniqueness of individuals’ experiences.

### Adaptation typologies using Q-methodology

Although many studies have examined IMIW’s physical and psychosocial distress and issues related to their adaptation in Korea [9, 18, 19], the research has mainly explored adjustment-related variables, placing little focus on adaptation patterns. Although typologies do not describe the entire experience of these women, they do provide information about potential issues impacting them. Q-methodology, which can generate patterns or typologies, has been used in many studies to inform both researchers and policy makers of the potential needs of immigrant populations. In addition, these typologies could be beneficial in developing and tailoring support programs for IMIW. Programs informed by qualitative data gathered from these women have the potential to improve the level of IMIW’s adaptation in Korean society, thus decreasing the consequences of physical and psychological distress and poor quality of life. The present study was conducted to fill this gap through Q-methodology, exploring Vietnamese IMIW’s adaptation types and conveying their unique, in-depth perspectives about their adaptation types. The study findings are expected to give healthcare providers and policy makers a better understanding of Vietnamese IMIW’s perspective on adaptation, and could thus contribute to helping Vietnamese IMIW stably and successfully settle in Korea.

### Purpose of the study

The purpose of this study was to identify a typology of subjective perspectives on adaptation to Korean culture and society among Vietnamese women living in rural areas, and to examine the characteristics of these groupings.

## Materials and methods

### Study design

This study was conducted using Q-methodology to identify and describe the subjective perspectives of Vietnamese IMIW on adaptation to Korean culture. Q-methodology is a research approach that allows investigators to explore people’s subjective perspectives, such as attitudes, beliefs, and values, regarding particular issues [20]. It combines the strengths of both qualitative methods (e.g., using interviews to examine person’s subjective experiences) and quantitative methods (e.g., using statistical techniques to reveal the structure of the subjective views reported) [21], and has been regarded as a useful strategy to examine subjective perspectives on nuanced or vague phenomena [22] as well as potentially controversial themes or topics [23]. The method can be conducted effectively with small samples, usually between 40 and 60 or less [24]. It is used in various fields including health research, and has potential in studies of health education and promotion [23]; it has been used in many studies to explore beliefs and attitudes on health, child rearing, and the differentiation of physical discipline and abuse [22, 25, 26]. Just as it has been used successfully in many other nursing studies, this methodology is appropriate for identifying the cultural adaptation of Vietnamese IMIW, the topic of the present study.

### Research procedure

#### Construction of the concourse (Q-population)

To develop the concourse, a research team consisting of four experts in Q-methodology and acculturation gathered IMIW’s views, opinions, and feelings regarding adaptation to Korean culture and society. Two trained nurse researchers reviewed the relevant literature and obtained self-referenced opinions about adaptation issues (e.g., life, discrimination) from nine Vietnamese IMIW through in-depth interviews. All interviews were conducted in Korean at the participants’ preferred location (e.g., the participant’s home), and were tape-recorded and transcribed to prevent data loss. The interviews lasted an average of 40 to 60 minutes. A Vietnamese translator was available during the interview to provide language assistance when necessary. Initially, 91 statements were extracted from the concourse data. After correcting for overlapping and ambiguous statements, 52 statements were generated as the second Q-population. The procedures for developing the Q-population were implemented by domain experts, including a Q-methodologist, three nursing professors, and a nurse researcher.

#### Construction of the Q-sample

Eliminating redundancies, 39 statements were selected as the final Q-sample representing the concourse (Table 1). The final statements were reviewed by a Korean bilingual teacher who taught Korean language to Vietnamese students. The verbatim statements were translated into Vietnamese by a Vietnamese IMIW translator who spoke both Vietnamese and Korean and who had lived in Korea for more than five years. The statements were back-translated into the Korean language by a Korean who spoke both languages. The original Korean version and the back-translated version were compared and evaluated to obtain a content validity index of .89. The statements were then numbered randomly and printed on 39 separate cards. To make the items easier for IMIW to understand, each double-sided card included the statement written in Korean on the front and in Vietnamese on the back.

**Table 1:** Q-statements.

QQQ Q-statements	
1. I am worried that I may make a mistake when speaking Korean.	21. I am familiar with the basics of the Korean culture and lifestyle (e.g., foods, customs, and games).
2. I am troubled because I am not able to express my feelings (e.g., angers and sorrows).	22. I feel that I am treated unjustly in Korea.
3. Misunderstandings occur frequently during conversations.	23. I regret that I cannot participate in social activities.
4. I am learning Korean from my husband’s family or through an educational program.	24. I feel more comfortable when I am with one of my countrymen than with a Korean.
5. I can speak Korean well.	25. I feel comfortable living in Korea.
6. I can read Korean well.	26. Currently I have a job.
7. I can write Korean well.	27. I get along better with Koreans than Vietnamese.
8. My husband and I often quarrel.	28. I get stressed because it is difficult for me to raise my children.
9. Sometimes I quarrel with my husband because we have different backgrounds.	29. I have different opinions than my family about raising children.
10. My husband forces me to have sex even when I do not want to.	30. I am worried that my children may be bullied at daycare.
11. The family finances are controlled by my husband or mother-in-law.	31. I participate in an educational program that focuses on raising children.
12. I often talk with my husband.	32. My family members help with child rearing.
13. My husband and I split the household chores.	33. I am satisfied with my parenting.
14. My family is the greatest support for me.	34. I sometimes regret that I married a Korean.
15. I have difficulties because the Korean culture and lifestyle are different from those of Vietnam.	35. My life is controlled by others.
16. I feel psychological pressure from being forced to unilaterally acculturate to the Korean culture.	36. I think that I am in charge of my life and lead it accordingly.
17. Sometimes I lose confidence and withdraw because Korean culture is very different from Vietnamese culture.	37. I can live a happy life if I work on it.
18. I try to know about Korean culture through self-help groups for Vietnamese people.	38. I am satisfied with life at present.
19. I enjoy Korean culture (e.g., food, games, and customs).	39. I consider myself a Korean national.
20. I frequently participate in leisure activities with my family.	

#### Selection of participants (P-sample)

A purposive sampling method was used to recruit participants. Vietnamese IMIW who attended local multicultural family support centers in two rural provinces, Cholla and Chungchung, were invited to participate in the study. These family support centers provide a variety of education programs and social activities to IMIW each year (e.g., Korean culture and etiquette, Korean language courses, parenting and child care, workshops for happy couples, cooking classes, health screening, job education, and physical activities, including social dance). Data were collected from 26 Vietnamese IMIW from May through August 2013. Participants were eligible for study if they (1) were over 18 years of age, and (2) could read, speak, write, and understand either Korean or Vietnamese. Considering that Q-methodology assesses within-individuals characteristics rather than looking for between-individuals differences [27], a small sample size was preferred; thus, the sample size in this study was deemed acceptable.

#### Q-sorting

**Fig 1:**
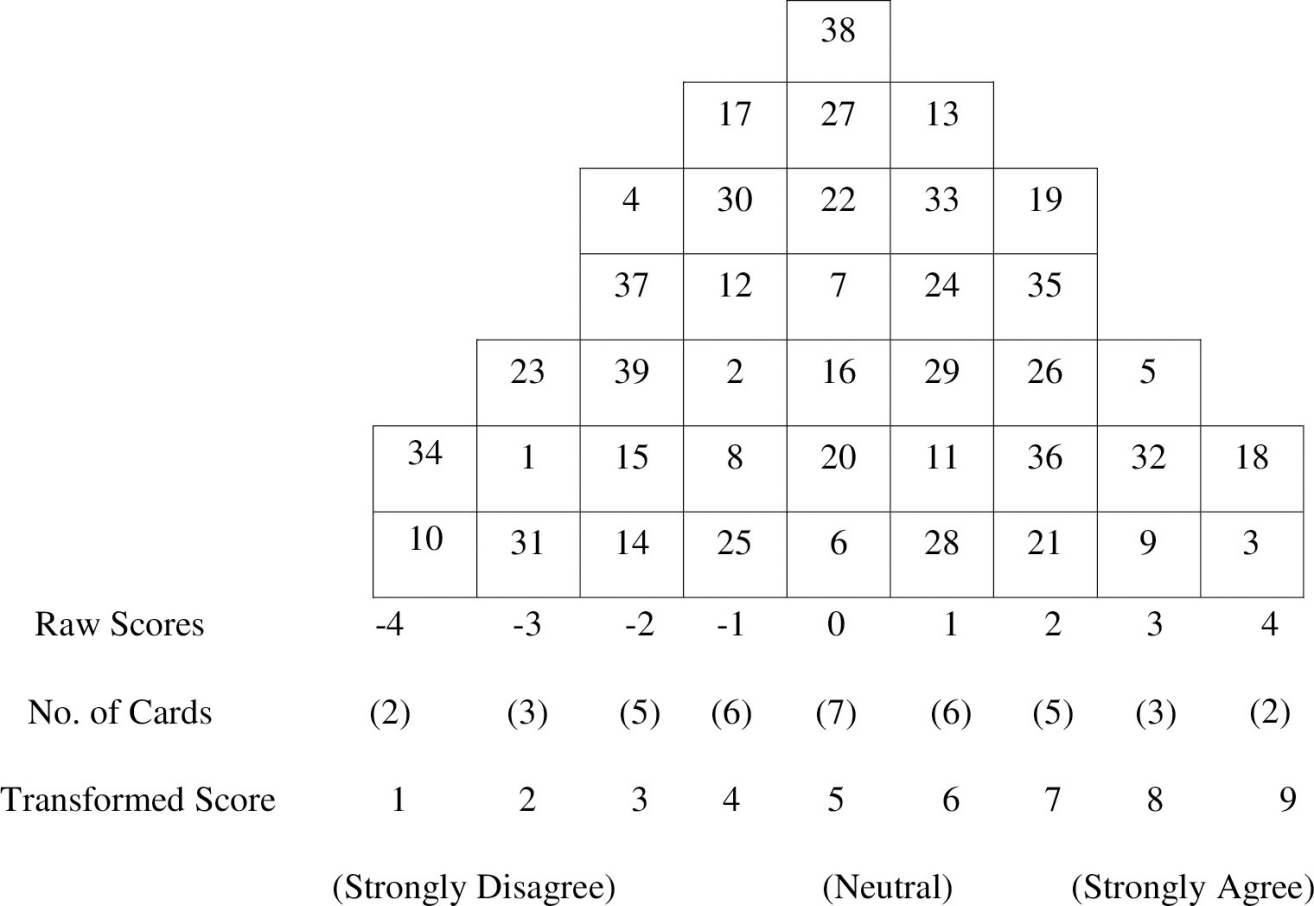
Sample of completed Q-sort table for rank ordering Q-sample.

Participants (the P-sample) were asked to read each statement and divide them into three piles according to their level of agreement with the statement (Agree, Disagree, Neutral), and to place the three piles in the Q-sorting table, which had a predesigned quasi-normal distribution labeled from *strongly disagree* at left to *strongly agree* at right, with *neutral* in the center (Fig 1). Participants were then asked to rank-order the three piles along the Q-sorting continuum. For example, a participant might place two statements most strongly agreed with in the +4 score section, three in the +3 score section, and so on, until all statements in the Agree pile were distributed between *agree* and *strongly agree*. The same process was used to distribute statements in the Disagree pile from *strongly disagree* to *disagree*. Finally, participants were asked to arrange the statements in the Neutral pile from *agree* to *disagree*. In each case, Q-sorting yielded a systematic forced distribution of the 39 Q-statements ordered on a scale from 1 to 9. After each Q-sort, participants were interviewed about the items they placed in the extreme columns, *strongly disagree* (-4) and *strongly agree* (+4). The interviews were conducted in Korean by researchers, and a Vietnamese translator was available during the classification and interviews to give language assistance when necessary. The final Q-sort was a matrix representing the participant’s operant subjectivity on the issue under consideration.

#### Data analysis

Principal components factor analysis using a pc-QUANL program was performed to reveal groupings in the data after each participant’s score was entered into the database. The following three points were relevant to analysis of the Q-sorts: obtaining Eigen values of at least 1.0 for the final interpretation, performing principle component analysis and varimax rotation to maximize the variance between each factor, and adopting Z-scores (standard scores) where statements with a Z-score above +1.0 were considered positive views and statements with a score below -1.0 were considered negative. A best estimate for each factor was calculated using factor weightings, demonstrating the prominence of an individual Q-sort in each factor. In addition, the Q-statements between a certain type and other types were compared to examine relative unique characteristics for each type.

### Validity and reliability

Four experts assured content validity by conducting a thorough review of the literature and evaluating the relevance and appropriateness of the Q-sample (content validity index > .80) [28]. To ensure face validity, the experts reviewed the Q-sample to edit for grammar and literacy issues and tried to maintain the participants’ original wording of statements. Reliability was established by repeated Q-sorting with a one-week interval. Test-retest reliability was acceptable, showing that the findings were consistent.

### Ethical considerations

This study was reviewed and approved by the Institutional Review Board of Woosuk University (WSOH IRB No. 1404–02). Participants were informed that the collected data would not be used for any purposes except this study, and that they could withdraw their participation at any time. Written informed consent was obtained from each participant.

## Results

In this study, all 26 participants were women with children (mean = 1.5). They ranged in age from 21 to 33 (mean age = 25.96 years) and had lived in rural provinces for an average of 2.8 years (range: 15–60 months) (Table 2). Subjective perspectives on adaptation to Korean culture and society were determined with factor analyses and revealed four types of adaptation that together accounted for 55.40% of variance: Type 1 (35.54%), Type 2 (8.33%), Type 3 (6.20%), and Type 4 (5.33%). The characteristics of each type were interpreted based on participants' demographic information, Z-scores of Q-statements and comments from participants as significant examples.

**Table 2:** Demographic characteristics of the P-samples.

	Full sample (n = 26)	Type 1 (n = 13)	Type 2 (n = 2)	Type 3 (n = 3)	Type 4 (n = 8)
Age (yrs)					
Mean (SD)	25.96 (3.03)	25.85 (3.41)	24.00 (1.41)	26.00 (4.58)	26.63 (2.20)
Range	21–33	21–33	23–25	21–30	24–29
Education (n, %)					
Middle school	1 (3.8%)	1 (7.7%)			
High school	14 (53.8%)	7 (53.8%)	2 (100.0%)	2 (66.7%)	3 (37.5%)
College	11 (42.3%)	5 (38.5%)		1 (33.3%)	5 (62.5%)
No. of child (n, %)					
1	14 (53.8%)	9 (69.2%)	1 (50.0%)	2 (66.7%)	2 (25.0%)
2	11 (42.3%)	3 (23.1%)	1 (50.0%)	1 (33.3%)	6 (75.0%)
3	1 (3.8%)	1 (7.7%)			
Length of stay (months) (n, %)					
15–23	7 (26.9%)	4 (30.8%)		2 (66.7%)	1 (12.5%)
24–35	8 (30.8%)	4 (30.8%)	2 (100.0%)	1 (33.3%)	1 (12.5%)
36–60	11 (42.3%)	5 (38.4%)			6 (75.0%)

### Type 1: Positive adaptation

Type 1, with Eigen value 9.284, was shared by 13 women and accounted for 35.54% of the total variance. On average, these women were 25.8 years old, had an average of 1.4 children, and had spent 2.7 years in Korea. Approximately 38.5% (n = 5) had completed college level education (Table 2).

Type 1 women agreed with Q-statements 12, 14, 21, 32, and 37. In contrast, the women did not agree with Q-statements 8, 10, 11, 26, and 34 (Table 3). Compared to participants of other types, Type 1 women had higher agreement (Z-score difference > 1) with following Q-statements 4, 21, and 25. Compared with other types, Type 1 showed higher disagreements (Z-score difference < -1) with the following statements, 17, 29, 30, and 34 (Table 4).

**Table 3:** Statements and array of Z-scores for each type and consensus items.

Type	Q statements*	Z-scores
1	12. I often talk with my husband.	1.64
	14. My family is the greatest support for me.	1.56
	37. I can live a happy life if I work on it.	1.31
	32. My family members help with child rearing.	1.20
	21. I am familiar with the basics of the Korean culture and lifestyle (e.g., foods and customs).	1.11
	8. My husband and I often quarrel.	-1.44
	10. My husband forces me to have sex even when I do not want to.	-1.49
	11. The family finances are controlled by my husband or mother-in-law.	-1.64
	26. Currently I have a job.	-1.93
	34. I sometimes regret that I married a Korean.	-1.96
2	14. My family is the greatest support for me.	1.89
	34. I sometimes regret that I married a Korean.	1.89
	12. I often talk with my husband.	1.70
	9. Sometimes I quarrel with my husband because we have different backgrounds.	1.34
	32. My family members help with child rearing.	1.34
	35. My life is controlled by others.	-1.27
	22. I feel that I am treated unjustly in Korea.	-1.27
	15. I have difficulties because the Korean culture and lifestyle are different from those of Vietnam.	-1.41
	38. I am satisfied with life at present.	-1.74
	7. I can write Korean well.	-2.10
3	29. I have different opinions than my family about raising children.	1.69
	2. I am troubled because I am not able to express my feelings (e.g., angers and sorrows).	1.59
	30. I am worried that my children may be bullied at daycare.	1.37
	14. My family is the greatest support for me.	1.35
	35. My life is controlled by others.	1.25
	19. I enjoy Korean culture (e.g., food, games, and customs).	-1.18
	10. My husband forces me to have sex even when I do not want to.	-1.26
	21. I am familiar with the basics of the Korean culture and lifestyle (e.g., foods and customs).	-1.49
	11. The family finances are controlled by my husband or mother-in-law.	-1.79
	26. Currently I have a job.	-2.05
4	14. My family is the greatest support for me.	2.50
	37. I can live a happy life if I work on it.	1.57
	39. I consider myself a Korean national.	1.24
	31. I participate in an educational program that focuses on raising children.	1.21
	36. I think that I am in charge of my life and lead it accordingly.	1.20
	24. I feel more comfortable when I am with one of my countrymen than with a Korean.	-1.30
	34. I sometimes regret that I married a Korean.	-1.45
	11. The family finances are controlled by my husband or mother-in-law.	-1.50
	35. My life is controlled by others.	-1.83
	10. My husband forces me to have sex even when I do not want to.	-1.83
Consensus items	22. I feel that I am treated unjustly in Korea.	-1.09
	8. My husband and I often quarrel.	-1.30

* Q-Statements with Z-scores greater +1.0 or less than -1.0

**Table 4:** Z-score differences between certain type and other types.

Type	Q-statements	Z-score Difference*
1	21. I am familiar with the basics of the Korean culture and lifestyle (e.g., foods and customs).	1.17
	4. I am learning Korean from my husband’s family or through an educational program.	1.11
	25. I feel comfortable living in Korea.	1.05
	30. I am worried that my children may be bullied at daycare.	-1.23
	29. I have different opinions than my family about raising children*.	-1.44
	17. Sometimes I lose confidence and withdraw because Korean culture is very different from Vietnamese culture.	-1.52
	34. I sometimes regret that I married a Korean.	-1.87
2	34. I sometimes regret that I married a Korean.	3.26
	11. The family finances are controlled by my husband or mother-in-law.	2.19
	26. Currently I have a job.	2.14
	10. My husband forces me to have sex even when I do not want to.	1.85
	39. I consider myself a Korean national.	-1.15
	23. I regret that I cannot participate in social activities	-1.19
	37. I can live a happy life if I work on it.	-1.37
	38. I am satisfied with life at present.	-2.23
3	35. My life is controlled by others.	2.61
	29. I have different opinions than my family about raising children.	2.17
	2. I am troubled because I am not able to express my feelings (e.g., angers and sorrows).	1.93
	23. I regret that I cannot participate in social activities	1.50
	17. Sometimes I lose confidence and withdraw because Korean culture is very different from Vietnamese culture.	1.16
	27. I get along better with Koreans than Vietnamese.	1.06
	4. I am learning Korean from my husband’s family or through an educational program.	-1.22
	12. I often talk with my husband.	-1.27
	19. I enjoy Korean culture (e.g., food, games, and customs).	-1.62
	26. Currently I have a job.	-2.05
	21. I am familiar with the basics of the Korean culture and lifestyle (e.g., foods and customs).	-2.30
4	1. I am worried that I may make a mistake when speaking Korean.	1.49
	38. I am satisfied with life at present.	1.33
	39. I consider myself a Korean national.	1.32
	31. I participate in an educational program that focuses on raising children.	1.13
	10. My husband forces me to have sex even when I do not want to.	-1.02
	24. I feel more comfortable when I am with one of my countrymen than with a Korean.	-1.13
	33. I am satisfied with my parenting.	-1.25
	35. My life is controlled by others.	-1.50

*Comparing with other types, Z-score differences with value greater +1.0 or less than -1.0

Participant number 5 who had the highest factor weight (2.13) in Type 1 was a 23-year old woman with one child and 2-year-long stay in Korea. She had completed high school education. She selected statements 12 and 14 and rated them highest agreement. She explained, “My husband teaches me Korean and Korean culture. Since I am married to a Korean man, I like my family in Korea the most. I rely on my husband greatly, and I am happy because I live with my husband.” She strongly disagreed with statement 34. Her reasons for this choice were expressed accordingly: “My husband is kind to me and he acts only after getting agreement from me. When my opinion is different from that of my husband, he usually yields. Our family relationship is good.”

Based on these viewpoints, Type 1 women viewed marriage to a Korean man as favorable and had positive attitudes toward their new life. In addition, they tried to embrace the cultural differences between the two countries and tried to become more familiar with Korean culture and lifestyle. In general, these women reported having a good relationship with their families and were adapting well to their new lives in Korea, having fewer conflicts of identity and culture. This type was classified as *positive adaptation*.

### Type 2: Passive adaptation with reservations

The Type 2 classification, with Eigen value 2.17, was shared by 2 women and accounted for 8.33% of variance. On average, these women were 24.0 years old, had completed high school, had one or two children, and had spent about 2.5 years in Korea.

Type 2 women agreed with Q-statements 9, 12, 14, 32, and 34. In contrast, the women did not agree with Q-statements 7, 15, 22, 35 and 38. Compared to participants of other types, Type 2 women had higher agreement with the following Q-statements: 10, 11, 26, and 34. Compared with other types, Type 2 showed greater levels of disagreement with the following statements: 23, 37, 38, and 39 (Table 4).

Participant number 26, who had the highest factor weight (1.83) in Type 2, was a 23-year old female with 2 children and had been in Korea approximately 3 years. She had completed high school education before coming to Korea. She selected the statements 12, 14, and 34 and assigned them the highest level of agreement. She explained: “My husband and mother-in-law help me with raising the children. I am happy living with my husband and children. However, my life is different from what I anticipated. Occasionally, it is hard to endure life; I feel a pressure on my chest. My husband taught me Korean so we often talk to each other.” She had the highest level of disagreement with statement 7 for the following: “I can write Korean only a little because the final consonants are so difficult.”

In summary, Type 2 women believed that their Korean families were a great support to them but they had experienced either marriage conflicts with their husband, communication problems due to the language barrier and/or complaints about receiving a limited amount of money. Overall, their experience was different from what they anticipated, and they were not currently satisfied with themselves or their situation. They might even regret their marriage but not openly protest it; thus, this type was classified as *passive adaptation* with reservations.

### Type 3: Adaptation with frustration

Type 3, with Eigen value 1.57, was shared by 3 women and accounted for 6.20% of the variance. On average, these women were 26.0 years old, had completed at least high school education, had either one or two children, and had spent 1.8 years in Korea.

Type 3 women agreed with Q-statements 2, 14, 29, 30, and 35. In contrast, the women did not agree with Q-statements 10, 11, 19, 21 and 26. Compared to participants of other types, Type 3 women had reported higher agreement with following Q-statements: 2, 17, 23, 27, 29, and 35. Compared with other types, Type 3 showed higher disagreement (Z-score difference < -1) with the following statements: 4, 12, 19, 21, and 26.

Participant number 19, who had the highest factor weight (1.13) in Type 3, was a 27-year old female with 2 children and had been in Korea about 3 years. She had completed a college level of education. She strongly agreed with statements 2 and 30. Her stated reasons were: “As I am not good at speaking Korean, I cannot express myself. No one understands my feelings. I feel depressed.” She also mentioned: “I am worried that my children may not have a friend because they cannot speak Korean well and have a different skin color.” She strongly disagreed with the statement 26. The reason she gave for this negative rating was “I am not working currently, though I want to work, because the children are so young and there is no one who can take care of them.”

Type 3 women were characterized as sometimes having trouble with their families due to differences in parenting beliefs and methods. They experienced internal stress, worrying that their children might be bullied at daycare or school. Moreover, they reported not only emotional distress but also social difficulties due to the language barrier, culture changes and lifestyle adjustment. They generally expressed a loss in self-confidence. Therefore, the classification of frustration adaptation was assigned to this type.

### Type 4: Adaptation and involvement

Type 4, with Eigen value 1.382, was shared by 8 participants and accounted for 5.33% of the variance. On average, these participants were 26.6 years old and had spent 3.4 years in Korea. The majority of them (n = 6, 75%) had two children. Approximately two-thirds (n = 6) had completed college level education.

Type 4 women agreed with Q-statements 14, 31, 36, 37, and 39. In contrast, these women did not agree with Q-statements 10, 11, 24, 34 and 35. Compared to participants of other types, Type 4 women had expressed higher agreement with following Q-statements: 1, 31, 38, and 39. Compared with other types, Type 4 reported higher disagreement with the following statements: 10, 24, 33, and 35.

Participant number 1, who had the highest factor weight (1.59) of Type 4, was a 28-year old female with 2 children and had been in Korea for 3 years. She had completed a college level education. She strongly agreed with the statements 14 and 39. She described the following reasons: “I am pleased to see my children growing up, and my husband understands my difficulties. I am a Korean because I am married to a Korean man.” She strongly disagreed with statement 10. She explained, “My husband and I have a good marital relationship, and he acts only after getting agreement from me.”

Summarizing the viewpoints above, Type 4 women were satisfied with their current situation living in Korea. In addition, they considered themselves Korean nationals. Believing that their lives were determined by their own efforts, they had a strong desire for self-realization through self-development. They showed increased engagement and participation in child-rearing activities and had a positive and active attitude towards speaking Korean correctly. Therefore, the title of *adaptation and involvement* was given to this type.

### Consensus statements

Participants of all 4 types shared similar attitudes and/or thoughts on adaptation regarding 2 items (Table 2). There was a consensus among the IMIW that they were treated relatively fairly in Korea and that their marital quarrels were infrequent.

## Discussion

This study provided information about the subjective perspectives of Vietnamese IMIW residing in rural Korea on adaptation to Korean society and culture. We reported the results of our Q-sort method analysis and included examples to support and clarify the classifications that were uncovered. The study’s findings revealed four types of adaptation with discriminating characteristics: Types 1 and 4 for positive perspectives, and Types 2 and 3 for negative perspectives. The commonalities and differences of each type are discussed below, beginning with positive perspectives and following with negative perspectives.

Type 1 (positive adaptation) and Type 4 (adaptation and involvement) women shared many common features. Both types were comfortable living in Korea, had a positive attitude, expressed beliefs that their own efforts could result in happiness, and could spend money independently. Overall, these women were young, had immigrated to Korea as soon as they were married, and gave birth soon after marriage. They received support from their husband and his family with respect to child care, language, and overall Korean lifestyle. This family support may have helped them feel accepted as a family member and relieved their child care burden, thus assisting in their positive adjustment to Korean life.

The characteristics of adaptation expressed by women of Types 1 and 4 indicated positive adaptation to Korean society and culture. These findings are consistent with previous studies reporting that social support including family support is a positive factor in adaptation to the new culture of a host country [12, 29] and improves IMIW’s marital satisfaction and psychological well-being [30, 31]. This is significant because IMIW with positive adaptation experiences might play an important central role in the family as mothers and wives, and could help their children adapt to Korean society and culture as well.

Compared to other types including Type 1, Type 4 women were more educated and had lived longer in Korea (more than three years), were better prepared for Korean society, and had found employment. Education, length of residence in the host country, and successful employment are significant factors in the adaptation process [31, 32]. Even more interesting, compared to Type 1, Type 4 women identified as Korean. These characteristics indicate that Type 4 women adapted to and felt comfortable being involved in Korean society and culture. This may be because they became Korean legally; according to the Korean Immigrant Act, IMIW can apply for Korean citizenship after maintaining their marriage in Korea for two years, and receiving the citizenship card takes considerable time. Having received the card, Type 4 women might experience a greater sense of belonging. Another possible explanation is giving birth. The average birthrate of Type 4 women (1.71) was higher than the total fertility rate of Korean women in 2017 (1.05) [33]. According to the findings of a qualitative review examining women’s experiences of maternal adaptation, giving birth was a turning point for IMIW, which led to rapid adjustment to Korean society to raise and support their children [34]. Thus, giving birth could have motivated the assimilation and involvement of Type 4 women, who were more likely to adapt to Korean society and culture voluntarily and actively.

Although the degree of acceptance varied, women of Type 2 (passive adaptation with reservations) and Type 3 (adaptation with frustration) expressed difficulties adapting to Korean society and culture. Their length of stay in Korea was relatively short, less than three years, which may not have been enough time to adjust to a new culture and society. Because they reside in rural areas, support programs available to urban women are less accessible to them [35, 36]. Poor transportation and limited access to government support programs present several challenges that may not be easily overcome. These women may experience greater challenges to adaptation than women in urban areas.

Unlike Types 1 and 4 women, Type 2 women showed signs of regretting their marriage to Korean men and dissatisfaction with their current situation; however, they did not directly express these attitudes. Data revealed that these women might be classified as achieving *passive adaptation*, meaning they attempted to adapt but experienced a degree of hesitancy and doubt. The main stressors for these women were conflict with their husbands, communication problems due to language barriers, and limited monetary allowance. Type 2 women may be unhappy with their daily lives when not getting along with their husbands [12, 37]. In addition, the language barriers experienced by Type 2 women might cause them to feel socially isolated. The literature states that if the circumstances are severe enough, such women might experience symptoms of anxiety and depression, leading to further problems in communicating with family members [11, 30]. Type 2 women expressed complaints about receiving a limited monetary allowance, despite currently having a job and earning an income. The present study did not ask about annual household income; however, limited household income could adversely affect satisfaction in international marriages [38].

Type 3 women, in contrast to Types 1 and 4, expressed problems adjusting to basic Korean culture and loss of self-confidence. Compared to Type 2, they reported conflicts with family members over child care. Type 3 women may experience isolation, gave birth soon after marriage, and are caring for a newborn; this combination of factors could result in psychological distress. Our findings suggested that Type 3 women were frustrated with pressures to adapt to Korean culture and society. Therefore, they need to overcome adaptation stress related to their new environment, new roles, and cultural awkwardness. It has been suggested that women like those of Type 3 would benefit from opportunities to interact with Koreans and other immigrants of their own nationality through in physical activities, self-help groups, and language courses in community centers [12, 29, 39]. An important consideration is that these programs should be offered by Vietnamese speakers, to overcome any communication problems.

Although a direct comparison is somewhat difficult because a different methodology was used, our study findings provide partial support for Berry’s acculturation model, which has four strategies: integration, assimilation, separation, and marginalization [16]. Similar to Berry’s model, our study also identified four types and revealed assimilation and separation strategies were. However, rather than separate integration and marginalization strategies, a mixed type was found in our study.

Types 1 and 4 found in this study are similar to the assimilation strategy. The difference between the two types is the degree of assimilation: Type 4 represents a state of complete assimilation and Type 1 an ongoing (or partial) state of assimilation. The existence of two distinct types of assimilation and the absence of integration types could be explained by the ethnically homogeneous society although the Korean government supports multiculturalism with national multicultural policy [40]. Such social atmosphere may present challenging circumstances for Vietnamese IMIW classified as Types 1 and 4, and may prevent women from retaining their heritage, culture, and identity, forcing them to assimilate into Korean culture and society. Type 2 is similar to the separation strategy, supporting the findings of Berry’s model [16]. The homogenous and male-centered social atmosphere of Korea, especially in rural areas, might result in resistance to assimilation and feelings of inequality, especially among women classified as Type 2, who immigrated to Korea on their own through brokered marriage.

Type 3 found in this study represents a non-classified type that requires more time and attention for classification; these women seem to be struggling to adapt, and exhibit mixed characteristics of both assimilation and separation. This finding is in line with previous studies that reported a mixed type with high levels of assimilation, marginalization, and/or separation [41, 42]. We found no marginalization strategy, in which immigrants neither maintained their heritage, culture, and identity nor had a relationship with the host country (Korea). This might be explained by the fact that our participants were recruited from community or public centers, which implies that they had some relationship with Korean society.

### Implications for nursing practice

The present study’s findings fill a gap in research on Vietnamese IMIW’s adaptation to Korean culture and society. The information is particularly important because it reports on women residing in rural areas, not urban areas, of Korea. It might be helpful to compare the typology and characteristics that emerged from our study and the various programs that are currently available to support women. The critical question is whether existing programs provided to urban women are sufficiently responsive and adequate for rural women, such as those in our study population. In addition, researchers and policy makers must focus on the role of language proficiency to ease IMIW’s transition into a new culture [43]. Cultural identity change is facilitated by (1) approval of and sympathy toward immigrants’ needs and challenges, (2) immigrant-specific social services (e.g., translation services and culturally syntonic mental health services), and (3) social policies to protect IMIW from discrimination [44].

In summary, healthcare providers and policy makers may choose to incorporate the information from our study to develop interventions and programs responsive to the needs of rural IMIW. Of particular value are the data on women’s spousal relationships and overall family support. This information can be used to develop educational materials, interventions, and social and health policies for incoming Vietnamese IMIW and their new family members in rural Korea. To help Type 1 women gain self-confidence similar to that found in Type 4 women, it might be advisable to augment current programs to incorporate employment and job search skills and coaching in obtaining Korean citizenship.

### Limitations

Although our study offers potentially important insights, there are also limitations to consider. First, we obtained a modest but adequate sample of IMIW; still, generalizations to all Vietnamese IMIW in rural Korea are not possible. Vietnamese IMIW in different geographical areas of Korea might have different experiences and perspectives than those found in our study, not only because of differences in residence but also because of differences in age, number of children, length of residence, and natal (pre-immigration) and current (post-immigration) socioeconomic status. The study findings should be interpreted cautiously due to limitations in their generalizability. Second, although the study design combines the strengths of both qualitative and quantitative research methodology, inferences about the factors that influence acculturation and adaptation stressors are tentative and need to be investigated further prior to drawing conclusions about what might constitute an effective family-sensitive adaptation support program for these women and their families. Third, this study focused on adaptation issues and analyzed the data according to the frame of reference of our IMIW sample. An alternative approach would be to group responses differently to reflect each of the women’s potential roles. For example, women could be asked to specifically describe their experiences with respect to the role functions of a wife, mother, and newly immigrated woman. Further, studies are needed to clarify the specific stresses women experience in each of these roles. Additionally, it would be important to explore the characteristics of IMIW’s husbands and their perspectives about adapting to marriage with their foreign brides. What stressors affect these husbands and what kind of support programs might they need. Future studies could focus on the couple as a unit and examine the support structures that would be helpful to them. Finally, the findings may reflect a selection bias, as the IMIW who were selected may have been more familiar with speaking and writing the Korean language. These individuals may have wanted to share their experiences, and were thus more likely to participate in this study.

## Conclusion

As revealed in this study, the experiences and perspectives of Vietnamese IMIW residing in rural Korea on adaptation are complex and differ across individuals. This study offers insights into the varied subjective perspectives of these Vietnamese IMIW. Use of Q-methodology facilitated our analysis of their experiences. To promote positive adaptation and minimize negative outcomes, healthcare providers and policy makers need to listen closely to the perspectives of these women and understand that their needs can be quite different. These findings, together with those of other descriptive studies, could be used to identify and validate useful adaptation models and typologies. Such models and typologies could guide the development and testing of tailored interventions and strategies to support the adaptation of rural immigrant Vietnamese women, and can highlight issues important to families as a whole.

## Supporting information

S1 Dataset (TXT)

## References

[1] Han S-H. A study on development of the Korea agricultural population forecasting model and long-term prediction. Journal of the Korea Academia-Industrial Cooperation Society. 2015;16(6):3797–806.

[2] Lee H, Williams L, Arguillas F. Adapting to marriage markets: International marriage migration from Vietnam to South Korea. Journal of Comparative Family Studies. 2016;47(2):267–88.

[3] Korea Research Institute for Local Administration. A study on improving the supporting system for married immigrant women delivered by local governments Seoul (South Korea): Korea Research Institute for Local Administration; 2008 [cited 2019 January 19]. Available from: http://www.krila.re.kr/publication/report/basic/615.

[4] Korea Research Institute for Local Administration. Improvement plan on policies for immigrants of local autonomous bodies for the settlement of multi-cultural communities. 2012 [cited 2019 January 19]. Available from: http://www.krila.re.kr/publication/report/basic/951.

[5] Korea Ministry of Government Legislation. Multicultural families support act, No. 15204 2017 [cited 2019 January 19]. Available from: http://www.lawnb.com/Info/ContentView?sid=L000B955DB6C8BC2_0_R10.

[6] Statistics Korea. Number of international marriage by countries. Daejeon (South Korea): Statistics Korea; 2018 [cited 2019 January 19]. Available from: http://www.index.go.kr/potal/main/EachDtlPageDetail.do?idx_cd=2430.

[7] Ministry of Agriculture F, and Rural Affairs,. International marriage status of rurual areas. Sejong, South Korea: Ministry of Agriculture, Food and Rural Affairs,; 2017 [cited 2019 January 19]. Available from: http://www.mafra.go.kr/woman/1195/subview.do.

[8] Korean Statistical Information Service. Divorce of foreign brides by countries Statistics Korea2018 [cited 2019 January 19]. Available from: http://kosis.kr/statHtml/statHtml.do?orgId=101&tblId=DT_1B85025&vw_cd=MT_OTITLE&list_id=MT_CTITLE_CD_10&scrId=&seqNo=&lang_mode=ko&obj_var_id=&itm_id=&conn_path=E1.

[9] Woo Y-H, Ha K-S. A study on the life satisfaction of migration women on international marriage. Journal of the Korea Academia-Industrial Cooperation Society. 2015;16(12):8535–49.

[10] Tsao Y, Creedy DK, Gamble J. Emotional well-being of Vietnamese immigrant women during the transition to motherhood: A descriptive cohort study. Nurs Health Sci. 2015;17(1):49–56. 10.1111/nhs.12143 24941901

[11] Uhm D. Effects on couples' communication, intimacy, conflict and quality of life by foot massage between immigrants. J Korean Acad Nurs. 2010;40(4):493–502. 10.4040/jkan.2010.40.4.493 20820116

[12] Yang JH, Park HJ, Kim SS, Kang EJ, Byun SH, Bang JS. Adaptation experience to family of immigrant women in multicultural families. J Korean Acad Nurs. 2012;42(1):36–47. 10.4040/jkan.2012.42.1.36 22410600

[13] Ahn JA, Kim T, Roh EH, Song JE. Health of international marriage immigrant women in South Korea: a systematic review. J Immigr Minor Health. 2018;20(3):717–28. 10.1007/s10903-017-0604-6 28584961

[14] Choi YJ. Mental health problems and acculturative issues among married immigrant women in Korea: A qualitative study. Women Health. 2016;56(6):713–29. 10.1080/03630242.2015.1118724 26605910

[15] Lee Y, Park S. The mental health of married immigrant women in South Korea and its risk and protective factors: A literature review. Int J Soc Psychiatry. 2018;64(1):80–91. 10.1177/0020764017744581 29231071

[16] Berry JW. Immigration, acculturation, and adaptation. Applied Psychology. 1997;46(1):5–34.

[17] Jones GW. International marriage in Asia: what do we know, and what do we need to know? Queenstown, Singapore: Asia Research Institute, National University Singapore; 2012.

[18] Hong SJ, Lee JM. Acculturation on stress, quality of life, and self-esteem in married immigrant women in Korea. International Journal of Bio-Science and Bio-Technology. 2016;8(3):77–84.

[19] Thao N, T. P.,. Different effects of acculturative stress and family life stress on depressive symptoms among married Vietnamese immigrant women in South Korea. Asian Social Work and Policy Review. 2016;10(2):225–36.

[20] Stephenson W. The study of behavior: Q-technique and its methodology. Chicago, IL: University of Chicago Press; 1953.

[21] Akhtar-Danesh N, Baumann A, Cordingley L. Q-methodology in nursing research: a promising method for the study of subjectivity. West J Nurs Res. 2008;30(6):759–73. 10.1177/0193945907312979 18337548

[22] Ho GWK, Gross DA. Pediatric Nurses' Differentiations Between Acceptable and Unacceptable Parent Discipline Behaviors: A Q-Study. J Pediatr Health Car. 2015;29(3):255–64. 10.1016/j.pedhc.2014.12.004 25620720

[23] Cross RM. Exploring attitudes: the case for Q methodology. Health Educ Res. 2005;20(2):206–13. 10.1093/her/cyg121 15385430

[24] Watts S, Stenner P. Doing Q methodology: theory, method and interpretation. Qualitative Research in Psychology. 2005;2(1):67–91.

[25] Cai D, Stone TE, Petrini MA, McMillan M. 'An exploration of the health beliefs of Chinese nurses' and nurse academics' health beliefs: A Q-methodology study'. Nurs Health Sci. 2016;18(1):97–104. 10.1111/nhs.12251 26727168

[26] Ha EH. Attitudes toward child rearing in female clinical nurses working in three shifts. Nurs Health Sci. 2016;18(4):416–24. 10.1111/nhs.12284 27098460

[27] Valenta AL, Wigger U. Q-methodology: Definition and application in health care informatics. Journal of the American Medical Informatics Association. 1997;4(6):501–10. 9391937 10.1136/jamia.1997.0040501 PMC61268

[28] Polit DF, Beek CT, Owen SV. Is the CVI an acceptable indicator of content validity? Appraisal and recommendations. Res Nurs Health. 2007;30(4):459–67. 10.1002/nur.20199 17654487

[29] Byoun SJ, Leung P. Understanding the cultural adaptation of foreign wives of South Korean men. Asia Pacific Journal of Social Work and Development. 2015;25(2):124–32.

[30] Chien LY, Tai CJ, Yeh MC. Domestic decision-making power, social support, and postpartum depression symptoms among immigrant and native women in Taiwan. Nurs Res. 2012;61(2):103–10. 10.1097/NNR.0b013e31824482b6 22307142

[31] Lin LH, Hung CH. Vietnamese women immigrants' life adaptation, social support, and depression. J Nurs Res. 2007;15(4):243–54. 18080969 10.1097/01.jnr.0000387621.95306.98

[32] Ho W-C, Cheung C-K. Ecological influences on Chinese migrant mothers' integration with Hong Kong. International Journal of Intercultural Relations. 2011;35:31–40.

[33] Statistics Korea. Changes of number of newborn babies and total fertility rate Daejeon (South Korea): Statistics Korea; 2018 [cited 2019 January 19]. Available from: http://www.index.go.kr/potal/main/EachDtlPageDetail.do?idx_cd=1428.

[34] Song JE, Ahn JA, Kim T, Roh EH. A qualitative review of immigrant women's experiences of maternal adaptation in South Korea. Midwifery. 2016;39:35–43. 10.1016/j.midw.2016.04.008 27321718

[35] Nam IS, Ahn S. Comparison of stress, social support, and marital satisfaction between married immigrant women in urban and rural areas. Korean Journal of Women Health Nursing. 2011;17(2):99–108. 10.4069/kjwhn.2011.17.2.99 37697559

[36] Kim HHS. Investigating the associations between ethnic networks, community social capital, and physical health among marriage migrants in Korea. Int J Env Res Pub He. 2018;15(1). 10.3390/ijerph15010147 PMC5800246 29342115

[37] Noda F, Noda M, Clark C. Family factors affecting adjustment in Japanese immigrant housewives. Canadian Journal of Psychiatry. 1990;35(8):689–92. 2282620 10.1177/070674379003500808

[38] Nho CR, Kim JH, Shin HJ, Heo SH. Meta-analysis of depression among marriage-based migrant women in South Korea. Asian Social Work and Policy Review. 2017;11(3):205–15.

[39] Chung HIC. Child rearing experiences of foreign wives married to Korean husbands. Asian Nurs Res. 2010;4(2):75–89. 10.1016/S1976-1317(10)60008-4 25030948

[40] Park J-D, Park J-H. A study of policy of multi-cultural society in Korea and suggestions for an advanced policy response. The Journal of Cultural Policy. 2014;28(1):35–63.

[41] Berry JW. Acculturation: living successfully in two cultures. International Journal of Intercultural Relations. 2005;29:697–712.

[42] Li Q, Oh I, Lee S-k. The relations of acculturation type, acculturation stress, basic psychological needs and college adaptation. Asian Journal of Education. 2016;17(1):101–20.

[43] Birman D, Simon CD. Acculturation research: Challenges, complexities, and possibilities. APA Handbook of Multicultural Psychology. 2014;1:207–30.

[44] Schwartz SJ, Montgomery MJ, Briones E. The role of identity in acculturation among immigrant people: Theoretical propositions, empirical questions, and applied recommendations. Human Development. 2006;49(1):1–30.

